# Service evaluation of the mental health assessment service (MHAS) in Dudley, West Midlands

**DOI:** 10.1192/bjo.2021.943

**Published:** 2021-06-18

**Authors:** Mark Winchester, Amitav Narula, Rachel Davis, Ella McGowan

**Affiliations:** Dudley and Walsall Mental Health Partnership NHS Trust

## Abstract

**Aims:**

To assess how well MHAS meets the service specification

To ascertain areas of good practice

To examine whether the referral form is being used in an appropriate manner

To elucidate areas of good communication and whether any improvement can be made

**Background:**

Launched in 2012, MHAS is the single point of access service for mental health services for patients aged 16–65 years, with a general practitioner (GP) in Dudley, who are not currently open to secondary care. Assessments are completed by a medic, community psychiatric nurse or jointly. It aims to identify the most appropriate care pathway for patients. This audit was a comprehensive assessment of how effective MHAS is at ensuring patients are adequately triaged.

**Method:**

10 cases from each month between April 2018 and March 2019 were randomly selected from all 980 anonymised MHAS referrals. A proforma was developed based on current practice, previous audits and service specification. A team of four doctors assisted in the data collection and only electronic health records (EHR) were reviewed.

**Result:**

88.3% of referrals were recorded on the EHR. Only 61.7% of referrals used the proforma with the other referrals mostly being in the form of a letter, which often missed out information vital to the triaging process. Only 4.2% of referrals are from Primary Care Mental Health Nurses (PCMHN) with 85.8% arising from GPs. Urgent referrals were not discussed with MHAS via telephone contact in about 60% of cases. The majority of patients had telephone screening completed the same day and were then discussed the next working day at the daily referral meeting. Although a brief summary for the GP was being sent the same day in all cases, over half of the comprehensive assessments were not being sent within the five day timeframe.

**Conclusion:**

All referrals must be uploaded to the EHR and completed using the service's proforma. PCMHNs may be currently under-utilised or effectively doing their jobs at managing mental health patients in primary care. GPs regularly referring via letter require further training and support to use the proforma. The proforma may require simplification to make it easier to complete. The service specification requires review as it makes unrealistic demands of the service. All referrals must be discussed at the daily referral meeting. Further investigation is required to understand why MHAS is struggling to meet timeframes for appointments and letters.

